# Essentials or Materia Medica, Therapeutics, and Prescription Writing

**Published:** 1906-02

**Authors:** 


ESSENTIALS OF MATERIA MEDICA, THERAPEUTICS, AND PRESCRIPTION WRITING. By Henry Morris, M.D., College of Physicians, Philadelphia. Seventh edition, thoroughly revised. By W. A. Bastedo, Ph. G., M.D., Instructor in Materia Medica and Pharmacology at the Columbia University (College of Phycians and Surgeons), New York City. 12mo, 300 pages. Philadelphia and London: W. B. Saunders & Company, 1905. Cloth, $1.00 net.

The student can not find a better or more practical work on Materia Medica, Therapeutics, and Prescription Writing than this little essentials from the press of W. B. Saunders and Company. But then, this work is no exception in this respect to all the other numbers of this excellent series of compends. Dr. Bastedo, in revising the book for this seventh edition, has brought it in accord with the new (1905) Pharmacopeia, introducing all the new remedies and carefully indicating their therapeutic doses and uses. For a work of three hundred pages it contains a mine of information so presented as to be easily grasped. We give it our unqualified endorsement.

